# Diagnostic and Therapeutic Approaches for Spinal Subarachnoid Hemorrhage Due to Spinal Aneurysms and Other Etiologies

**DOI:** 10.3390/jcm14072398

**Published:** 2025-03-31

**Authors:** Biyan Nathanael Harapan, Robert Forbrig, Thomas Liebig, Christian Schichor, Jun Thorsteinsdottir

**Affiliations:** 1 Department of Neurosurgery, LMU University Hospital, LMU Munich, 81377 Munich, Germany; biyan.harapan@med.uni-muenchen.de (B.N.H.);; 2 Institute of Neuroradiology, LMU University Hospital, LMU Munich,81377 Munich, Germany

**Keywords:** spinal aneurysm, subarachnoid hemorrhage, SAH, spinal subarachnoid hemorrhage, clipping, neurosurgery, vascular disorder, vascular pathology

## Abstract

**Background**: Spinal subarachnoid hemorrhage (sSAH) is a very rare disease. Detailed information about the natural course, pathogenesis, radiological manifestation, and therapeutic management is lacking. This study aimed to analyze patients diagnosed with sSAH, focusing on the origin, management strategies, and therapeutic approaches to sSAH. **Methods**: The study included a cohort of patients admitted to the Department of Neurosurgery, LMU University Hospital, LMU Munich, between January 2021 and December 2024 with a confirmed diagnosis of spinal subarachnoid hemorrhage and, among other things, spinal aneurysms. Data on the included patients were recorded with emphasis on demographics, radiological examination (CT, MRI, and DSA), aneurysm-specific characteristics, and clinical outcome. **Results**: The study included six patients diagnosed with spinal subarachnoid hemorrhage via multimodal imaging. The etiology of sSAH was identified in all cases, encompassing spinal aneurysms in three patients, anticoagulation therapy in two cases, and bony microspurs in one case, with management strategies tailored as either conservative (monitoring and imaging) or surgical (aneurysm resection, arterial feeder coagulation, or evacuation of intraspinal bleeding). No major adverse events were observed, and all the patients demonstrated neurological improvement or exhibited only mild-to-moderate disability during follow-up. **Conclusions**: Spinal subarachnoid hemorrhage can be due to a ruptured spinal aneurysm, but in some cases, other underlying causes should be considered as the source of the hemorrhage. Given the scarcity of literature on this condition, it is crucial to identify the correct diagnosis and implement a patient-tailored therapeutic approach.

## 1. Introduction

Spinal subarachnoid hemorrhage (sSAH) is a very rare disease caused by a diverse range of underlying conditions, potentially resulting in severe neurological deterioration. Ruptured spinal aneurysms as a potential origin are very rare, and until now, only few case reports and series have described this entity. Spinal aneurysms can be associated with arteriovenous malformations/fistulas [1,2] or other vasculopathies provoking hemodynamic stress, e.g., aortic coarctation, bilateral vertebral occlusion [3], or dissection caused by NDMA consumption [4]. In the case of aortic coarctation, it is assumed that the anterior spinal artery can be a significant route for collateral circulation [5]. In addition, other diseases such as polycystic kidney disease [6] or Behcet’s disease [7] have been reported to be associated with spinal aneurysms. However, the difference between spinal and common intracranial aneurysms is that the former usually do not occur in arterial bifurcations [8].

In cases of SAH resulting from aneurysms in the craniocervical junction, patients commonly display symptoms such as headaches, meningism, and neurological deterioration comparable to those observed in ruptured intracranial aneurysms [9,10]. In cases of ruptured aneurysms localized in the thoracic or lumbar regions, patients may present with intense back pain, sensory or motor deficits, or abdominal symptoms [11,12]. Consequently, diagnosing this rare disease can be challenging and delayed due to the diverse and diffuse nature of the symptoms.

The standard diagnostic approach involves spinal angiography. However, when a selective spinal angiogram is unavailable, additional examinations such as magnetic resonance imaging (MRI) or CT angiography can offer valuable diagnostic information. Furthermore, in situations where the aneurysms are diminutive or exhibit partial thrombosis, identifying the precise source of bleeding becomes challenging, requiring repeated multimodal imaging [6].

Numerous recommendations for the therapeutic approach to spinal aneurysms are discussed in the literature, encompassing surgical excision, wrapping, interventional occlusion, or conservative therapy, depending on the specific localization and vascular anatomy [13,14,15]. Given the rarity of this condition, there is a lack of established protocols for determining the appropriate timing and approach for the treatment of intraspinal aneurysms.

Here, we present six cases of primary spinal SAH, the majority being due to a ruptured spinal aneurysm, with special attention to the diagnostic approach and therapeutic management. As detailed case evaluations on spinal SAH and spinal aneurysms are very rare and the current literature is scarce, we further conducted a comprehensive review with a special focus on the latest studies on the clinical presentation, management approaches, and outcomes.

## 2. Materials and Methods

This single-center study involved a retrospective analysis of all patients diagnosed with spinal subarachnoid hemorrhage (sSAH) at the Department of Neurosurgery, LMU University Hospital, Munich, Germany, between January 2021 and December 2024. Cases involving traumatic subarachnoid hemorrhages and extensive subarachnoid hemorrhages resulting from the rupture of intracranial aneurysms that extended into the spinal canal were excluded. The rationale for this exclusion criterion was to maintain a focused analysis on sSAH originating primarily from spinal pathologies, ensuring the distinction between the hemorrhages caused by cranial events with secondary spinal involvement and those originating directly within the spinal axis. Patient data including demographic information, radiological findings from CT, MRI, and digital subtraction angiography (DSA), as well as specific aneurysm characteristics, including location and morphology, were analyzed. MRI images were obtained using a GE Signa 3 Tesla scanner. Clinical outcomes, including neurological status (according to the modified Rankin scale) and management strategies, were also evaluated to provide a comprehensive understanding of each case.

Additionally, a systematic literature review was performed using the PubMed database. The search focused on English-language publications available from January 2021 through December 2024. The following key terms were employed to identify relevant studies: “spinal”, “subarachnoid hemorrhage”, “SAH”, “aneurysm”, and “spinal aneurysm”. Two independent reviewers (B.N.H. and J.T.) systematically screened the titles and abstracts of all the retrieved articles to assess their relevance. Articles were excluded if they did not primarily address spinal subarachnoid hemorrhage. The final selection of studies provided a comprehensive overview of recent advances and insights into the etiology, diagnosis, and management of sSAH.

This study was conducted in compliance with the ethical principles outlined in the 1964 Declaration of Helsinki and its subsequent amendments. Informed consent was obtained from all the individual participants involved in the study.

## 3. Results

### 3.1. Case 1

A 47-year-old female patient was admitted to our emergency department due to progressive cephalgia over several days. The pain, localized in the back and radiating to the shoulders, prompted a cranial CT, revealing a Fisher IV SAH with concurrent spinal SAH extending from C1 to T3. In response to her loss of consciousness, the patient was intubated, and an external ventricular drainage (EVD) was placed.

**Figure 1:**
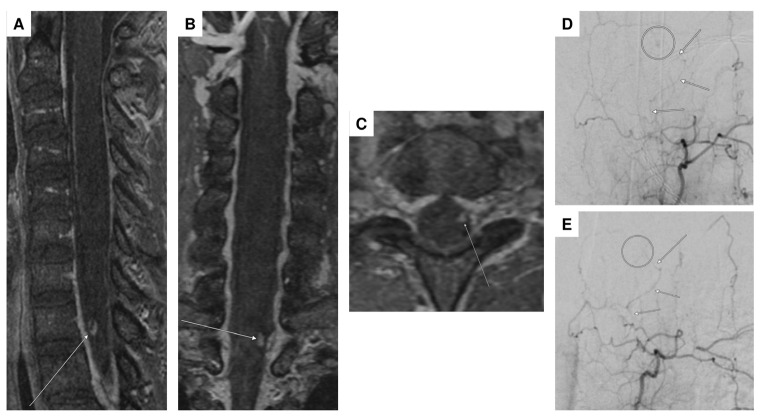
High-resolution contrast-enhanced MR angiography (ceMRA extreme) demonstrated a probably partially thrombosed spinal aneurysm (arrows) perimedullary on the left with a diameter of 4 mm at the T1 vertebral level ((**A**–**C**): sagittal, coronal, and transversal planes). DSA in the anteroposterior plane confirmed the partially thrombosed spinal aneurysm at the T1 vertebral level (circle), arising from the radiculomedullary T3/4 artery on the left (arrows) (**D**). Follow-up DSA was performed after 3 months, visualizing the same arterial supply (left T3/4, arrows) without evidence of residual aneurysm perfusion consistent with complete (spontaneous) aneurysm occlusion (**E**).

DSA including spinal vessels was subsequently conducted, revealing an aneurysm at the T1 level arising from the left T3/4 radiculomedullary artery (Figure 1D). Additional magnetic resonance angiography (MRA) demonstrated progressive partial thrombosis of the aneurysm (Figure 1A–C). Therefore, the immediate need for surgical or interventional occlusion was not indicated. Prophylactic administration of nimodipine and transcranial ultrasonography were implemented to exclude vasospasm. After continuous monitoring at the intensive care unit (ICU) for 30 days, the patient successfully underwent a gradual weaning process from ventilation and the EVD. A follow-up MRA after one week revealed complete thrombosis of the aneurysm. Since the non-invasive MRA provided clear evidence of aneurysm thrombosis, a follow-up DSA was not deemed necessary.

The patient did not present any neurological deficits (mRS = 1) and was discharged after a 30-day hospital stay to rehabilitation. Follow-up DSA after 3 months (Figure 1E) and spinal MRI after 6 months continued to demonstrate complete occlusion of the aneurysm.

### 3.2. Case 2

The patient presented with intermittent hypo-/paresthesia in bilateral lower extremities and myofascial pain syndrome. Neurological assessment revealed no abnormalities.

**Figure 2:**
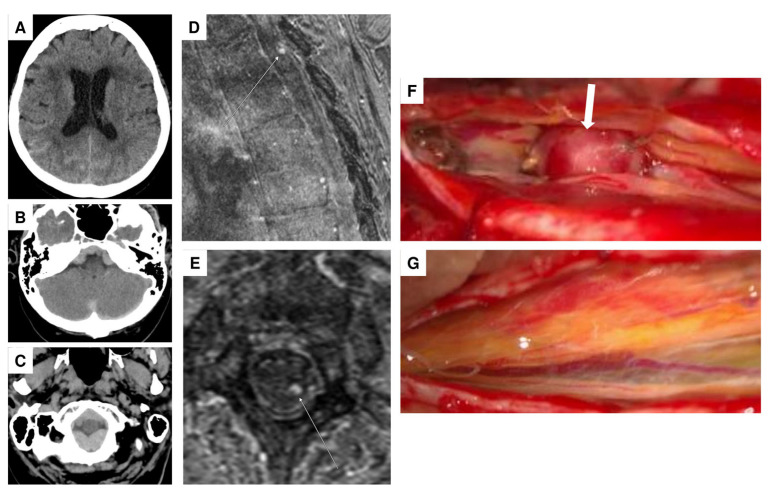
Noncontrast head CT in the transversal plane showing minor subarachnoid hemorrhage, primarily in the right temporoparietal region (**A**), and distinct subdural hemorrhage in the posterior fossa, particularly involving the craniocervical junction (**B**,**C**). High-resolution contrast-enhanced MR angiography (ceMRA extreme) in the sagittal (**D**) and transversal (**E**) planes, showing a suspected perimedullary aneurysm dorsolaterally on the left at the T2 level (arrows), measuring approximately 3 mm in diameter. Intraoperative visualization of the aneurysm ((**F**), arrow). Post-resection view following coagulation of the feeding artery, showing no residual aneurysm (**G**).

A 69-year-old female patient was admitted to the emergency department of a referral hospital with abdominal and thoracic pain, accompanied by pollakisuria, obstipation, and hypotonia. An initial abdominal CT ruled out aortic dissection, free fluid in the abdomen, and ileus. During the assessment of vital parameters, the patient experienced a hypertensive crisis, reaching a systolic blood pressure of 246 mm Hg. Cardiac involvement was excluded through examinations by internal medicine colleagues. The neurological examination revealed meningism and a positive Lasègue sign, lacking sensorimotor deficits. Subsequent CT imaging revealed a minor SAH, but also an acute subdural hematoma predominantly located at the foramen magnum and extending throughout the entire spinal canal (Figure 2A–C), with lumbar puncture results indicating a significant erythrocyte count of 340,000/µL.

The patient was then transferred to the neurosurgical intensive care unit, where extensive imaging, including multiple CT, MRI, and angiography studies, failed to identify the source of bleeding. After 13 days, the patient presented with altered consciousness and a hydrocephalic component on a cranial CT, leading to the implantation of a ventriculoperitoneal (VP) shunt after the initial placement of an EVD catheter and unsuccessful weaning from the EVD. Following this intervention, the patient exhibited gradual improvement in vigilance and reduced cephalgia. A subsequent MRI performed 16 days later, following partial resorption of the spinal SAH, revealed the presence of an intraspinal extramedullary aneurysm at the T2 level (Figure 2D,E). A subsequent DSA failed to confirm the aneurysm. However, an interdisciplinary neurovascular conference recommended surgical exploration.

During the surgical exploration involving a Th 2 laminectomy, an intradural extramedullary aneurysm was identified and dissected from the Th 2 nerve roots (Figure 2F). Temporary clips were applied to the parent arteries; we then evaluated for changes in neurophysiological monitoring to identify whether complete resection would lead to ischemic complications. No abnormalities were detected by neurophysiological monitoring during the five minutes of temporary clipping, allowing for a complete resection of the aneurysm. The feeding artery was coagulated, and the aneurysm was resected (Figure 2G). Intraoperative monitoring, including SSEP and MEPs, revealed no abnormalities. Histological analysis confirmed the presence of a partially thrombosed aneurysm, which may account for its absence on angiography due to reduced or absent blood flow within the lesion.

The patient was extubated without any motor deficits and was subsequently transferred to a neurorehabilitation center. The last follow-up occurred 2.5 years after the onset of the initial symptoms. The patient exhibited stable gait ataxia with an mRS score of 2.

### 3.3. Case 3

A 25-year-old female patient developed acute cephalgia and hypesthesia across her entire body. Upon the arrival of the emergency physician, the patient presented with tetraplegia and asystole, necessitating a resuscitation effort lasting four minutes. Subsequently, she was transferred to a paraplegic center. The initial MRI showed an intraspinal intramedullary bleeding extending from the medulla to C6. Following six days of ICU management, the patient’s cardiopulmonary stability allowed for extubation.

**Figure 3:**
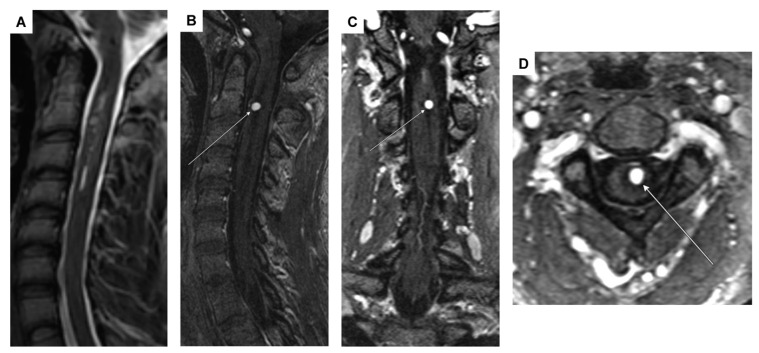
MRI showing a 4 mm intramedullary arterial aneurysm (arrows) with homogeneous contrast enhancement at the C2 vertebral base level and associated spinal cord edema/malacia from C2 to C4, likely post-hemorrhagic in origin. (**A**) Sagittal T_2_-weighted sequence; (**B**–**D**) arterial-phase contrast-enhanced MR angiography in the sagittal, coronal, and transversal planes.

Remarkably, the initial tetraplegia exhibited improvement through rigorous neurorehabilitation efforts, enabling the patient to achieve a mobility of 200 m with crutches. A follow-up MRI, conducted four weeks post-resorption of the intramedullary bleeding, revealed a contrast-enhancing intramedullary lesion at C2. This lesion was identified as an intramedullary aneurysm, possibly originating from a side branch of the anterior spinal artery (Figure 3A–D).

**Figure 4:**
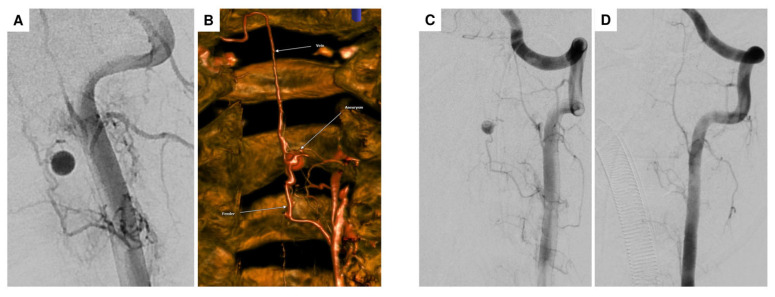
DSA in the anteroposterior plane showing the intramedullary arterial aneurysm at the C2 level, associated with an arteriovenous (AV) shunt originating from an arterial feeder of the left vertebral artery at the C3/4 level, consistent with a perimedullary AV fistula (**A**). Flat-panel CT angiography with volume rendering technique (VRT) reconstruction clearly shows the AV fistula morphology, including the arterial feeder (bottom arrow), the intramedullary aneurysm (middle arrow), and the draining shunt vein (top arrow) (**B**). Compared to preoperative DSA (**C**), no residual aneurysm or shunt vein was observed postoperatively, confirming complete occlusion of the AV fistula (**D**).

**Figure 5:**
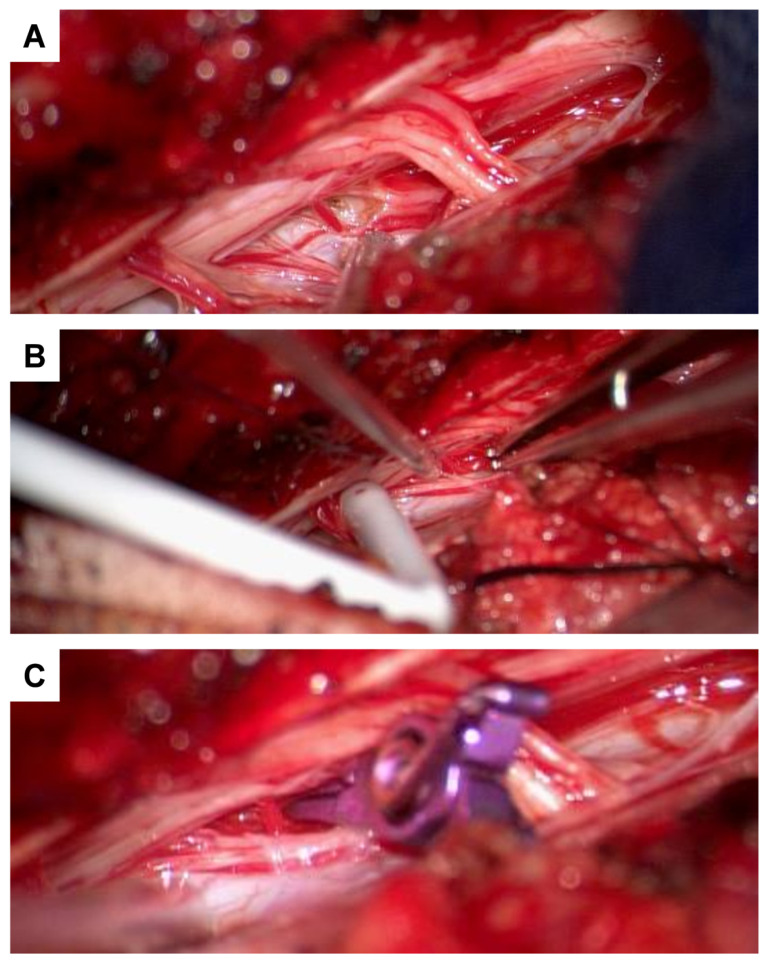
Intraoperative images demonstrating arterial feeder management. Suspected arterial feeder was identified laterally (**A**). Due to its intramedullary location, the aneurysm was not directly visualized to minimize perioperative morbidity. The use of a micro-Doppler probe allowed identification of the aneurysm (**B**). Temporary clipping of the feeder was performed (**C**), followed by intraoperative monitoring (IOM), which remained stable. Doppler assessment indicated no remaining signal, allowing for safe coagulation and transection of the arterial feeder.

For further diagnostic exploration and therapeutic intervention, the patient was then transferred to our hospital. Angiography detected a perimedullary fistula with an associated aneurysm at C2, fed by a radiculomedullary artery arising from the left vertebral artery at the C3/4 level (Figure 4A–C). Attempting interventional occlusion was challenging due to potential flow reversal and incomplete occlusion. Therefore, surgery proceeded. A C3 hemilaminectomy and durotomy were performed (Figure 5A). Subsequent ultrasonography and micro-Doppler could localize the aneurysm intramedullarily in the anterior part of the spinal cord (Figure 5B). Given the potential for severe morbidity from dissecting the aneurysm in the anterior part of the spinal cord, verification of aneurysm occlusion was exclusively achievable through ultrasonography and micro-Doppler (a clear signal was detected when inspecting the spinal cord). The lateral radicular vessels were explored, revealing an atypical vessel, which was carefully dissected and closed with forceps. Micro-Doppler was repeated in the suspected aneurysm area, but the signal was no longer detectable as the suspicious vessel was occluded. Intraoperative neuromonitoring (IONM) remained stable. Then, the suspicious radiculomedullary artery was occluded with a hemoclip, and IONM remained stable, confirming that the suspicious artery was not a vital spinal cord vessel, so its occlusion would not pose any risk of ischemic complications (Figure 5C). The radiculomedullary feeder was coagulated and cut. Micro-Doppler and ultrasonography confirmed the absence of a signal in the suspected aneurysm area. The neurological examination at discharge on the 8th postoperative day revealed mild-to-moderate muscle weakness, particularly on the left side (strength 3–4/5).

During the last follow-up three years later, the patient presented with a residual left-sided hemiparesis (mild in the left upper extremity (strength 4/5) and mild-to-moderate in the left lower extremity (strength 3–4/5)), accompanied by allodynia and gait ataxia (mRS = 3).

### 3.4. Case 4

**Figure 6:**
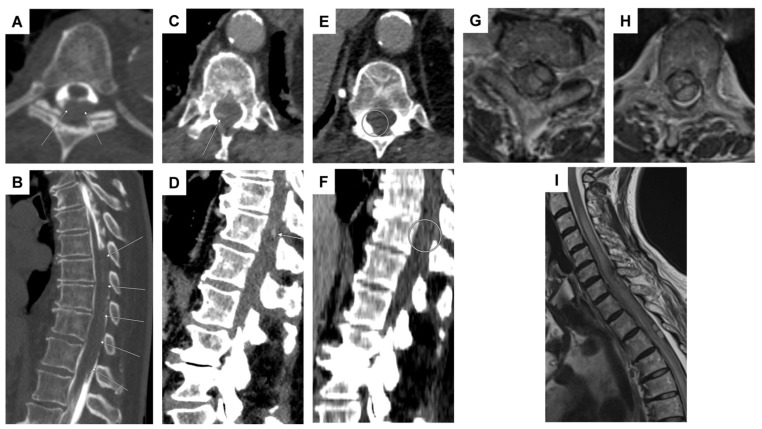
Case 4: imaging series illustrating suspected thoracic sub-/intradural bleeding and contrast-enhanced findings potentially indicative of active hemorrhage. CT myelography ((**A**,**B**): transversal and sagittal planes) showed evidence of an extensive dorsal sub-/intradural hemorrhage of the thoracic spine (arrows). Transversal (**C**) and sagittal (**D**) planes of a body CT scan in the portal-venous phase revealed a streak-like spinal contrast enhancement at the T11 vertebral level, located most likely perimedullarily and dorsolaterally on the right (arrows). This finding, considered a potential “spot sign,” suggested possible active spinal bleeding, likely related to apixaban therapy. Nine days later, a follow-up CT scan in the portal-venous phase ((**E**,**F**): transversal and sagittal planes) did not reveal signs of active spinal bleeding any longer, as the suspicious contrast-enhanced lesion at the T11 level had disappeared (circled region). Note: an initial thoracic and lumbar DSA did not reveal any vascular pathology (not shown). Case 5: transversal planes at the levels of C7 (**G**) and T3 (**H**), alongside a sagittal view (**I**) on an MRI T_2_-weighted sequence, demonstrate a space-occupying intradural hematoma extending from C6 to T3 on the left side. The hematoma exhibited a crescent-shaped configuration, displacing and compressing the spinal cord to the right both ventrally (**G**) and dorsally (**H**). Additionally, subarachnoid hemorrhage components extended caudally, and a long-segment myelopathy was evident, spanning from C3 to T6.

An 86-year-old female patient presented at the emergency department of a referral hospital with position-dependent sacroiliac pain. Subsequently, there was a sudden onset of paraparesis of the legs and hypesthesia below Th 10. Over the following hours, these symptoms gradually improved, leaving residual gait instability and paresthesia in the right leg. In the cranial CT and CT angiography, there was no evidence of bleeding, ischemia, or stenosis. An incidental 4 × 10 mm aneurysm on the right intradural ACI was detected. Metal artifacts were observed on the left side due to the history of clipping of an intradural ACI aneurysm on the left side. Doppler/duplex sonography revealed a pronounced macroangiopathy extracranially, with no hemodynamically relevant stenoses either extracranially or intracranially. Aortic CT angiography and spine CT were unremarkable. A cranial MRI could not be conducted due to the presence of the intracranial metal clip (material unknown). Forty-eight hours after discontinuing oral anticoagulation (apixaban), a lumbar puncture revealed evidence of a SAH. Subsequent myelography, followed by post-contrast CT myelography, revealed extensive subarachnoid blood accumulations in the cervical and thoracic spine, most pronounced ventrally to the upper cervical cord and dorsally to the lower thoracic cord (Figure 6A,B), without identifying the source of bleeding.

The patient was subsequently transferred to the stroke unit for cardiovascular and neuromonitoring and further diagnostic evaluations. DSA and multiphase CTA failed to detect any evidence of a vascular pathology. In comparison with the previous CT imaging (Figure 6C,D), the round, striped contrast enhancement at the level of T10/11 was no longer identifiable (Figure 6E,F). Given the lack of space-occupying hemorrhage and the overall clinical and neurological improvement, surgery was deemed unnecessary.

Following the thorough diagnostic evaluation, the patient was subsequently transferred back to the original referring hospital as part of her ongoing medical care. The follow-up examination 4 months later revealed that the patient still exhibited gait instability, with no evidence of manifest or latent paresis or sensory disturbances. Additionally, the patient was utilizing a walking stick on the right side (mRS score = 3).

### 3.5. Case 5

A 73-year-old patient initially presented with acute headaches and subsequently contacted emergency services. Upon arrival at a referral hospital, she was found to have a hypertensive crisis, with systolic blood pressure reaching 200 mm Hg. Further diagnostic workup revealed a bihemispheric supra- and infratentorial SAH. Due to progressive neurological decline manifesting as paraparesis of the legs, she was transferred to our clinic for advanced diagnostics and therapy. On admission, severe paraparesis of both legs (progressive since the previous evening) and exacerbated back pain were noted. A CTA of the thoracic and abdominal aorta ruled out aortic dissection but revealed a left ventricular pseudoaneurysm and a known atrial thrombus. Anticoagulation with rivaroxaban was paused. Subsequent spinal MRI revealed a space-occupying hemorrhage in the lower cervical and upper thoracic spine (Figure 6G–I). DSA did not identify a clear vascular pathology. The patient underwent an emergency laminectomy at the T2/3 level, followed by intradural hematoma evacuation for decompression, with the maximum site of bleeding identified at the T3 level, where intradural hemorrhage caused myelopathy and spinal cord compression. Postoperatively, the paresis did not improve markedly. Follow-up MRI and DSA again did not identify a clear bleeding source. As this case is very recent, no further follow-up information is available.

### 3.6. Case 6

A 55-year-old patient initially presented at a referral hospital with cervicothoracic pain after lifting a shopping bag and subsequent voiding dysfunction. A CT scan revealed an epi-/subdural and subarachnoid hematoma in the spinal canal, spanning from C6 to T5, which was surgically relieved. Follow-up imaging disclosed a progressive enlargement of the epidural hematoma and the emergence of a left-sided myelopathy at the T1/2 level. Consequently, the patient was transferred to our clinic for further investigation of the source of bleeding. A repeated surgical procedure was initially postponed in favor of further detailed diagnostics, given the absence of focal neurological deficits (such as sensory disturbances or paresis).

**Figure 7:**
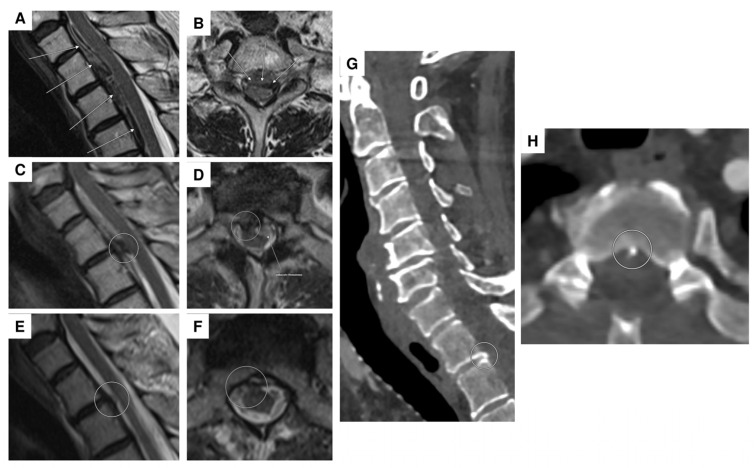
Spinal MRI of acute and subacute epi-/subdural hemorrhage with a suspected thrombosed perimedullary aneurysm at the T1/2 level. Initial MRI showed an acute epi-/subdural hemorrhage extending ventrally from levels C1 to T4, with maximal prominence at level T1 (arrows; T_2_-weighted sequence in the sagittal (**A**) and transversal planes (**B**)). The follow-up MRI five days later ((**C**): sagittal T_2_-weighted sequence; (**D**): transversal contrast-enhanced T_1_-weighted sequence) revealed ongoing (subacute) epi-/subdural bleeding from levels C7 to T3 ventrally (the arrow in (**D**)) and a newly suspected thrombosed aneurysm with slight wall enhancement at the T1/2 level, located perimedullarily and ventrolaterally on the right (circles in (**C**,**D**)). Follow-up MRI two weeks after the initial scan ((**E**,**F**): sagittal and transversal T_2_ -weighted sequence) revealed regression of the hematoma, with no remaining evidence of the aneurysm, suggesting probably complete occlusion (circled area). Spinal CT demonstrated a bony spur (circled area) at the dorsal margin of the intervertebral T1/2 level ((**G**,**H**): sagittal and transversal planes). Note: two DSAs were performed, with no vascular pathology detected.

In-house spinal MRI demonstrated a lesion suspicious for a spinal aneurysm at the T1/2 level. Comparable to the external MRI, a persistent, predominantly subdural, partially epidural, and subarachnoid bleeding in the upper thoracic region was detected. This bleeding was localized predominantly ventrally of the spinal cord, starting at the vertebral body of C7 and extending caudally to the vertebral body of T4 (Figure 7A,B). In addition, when considering the native cranial CT alongside the previous findings, there was a strong suspicion of ventral cervicothoracic and dorsal midthoracic subarachnoid bleeding. Furthermore, a 6 mm T_2_ hypointense round lesion was located on the right anterolateral part of the spinal cord at T1/2 (Figure 7C,D). Considering the blood distribution pattern, this was most suspicious for a thrombosed (ruptured) perimedullary intraspinal aneurysm.

With the progressive hematoma extending towards the occipital region and causing an escalating impairment of consciousness, the patient was transferred to our neurosurgical intensive care unit for continuous monitoring and therapeutic intervention. Upon admission, the patient was conscious (GCS 13), showed motor response in all extremities, and pupillary motility was unremarkable. Notably, the patient presented with meningism. After admission to the intensive care unit, there was a progressive decline in vigilance, tentatively attributed to metabolic derangement. Considering the patient’s pre-existing SGLT-2 inhibitor therapy (empagliflozin), a history of extended fasting, dehydration due to nausea and vomiting, a provisional diagnosis of euglycemic diabetic ketoacidosis under SGLT-2 inhibitor therapy was established. The patient displayed increasing polyuria indicative of cerebral salt-wasting syndrome, which was managed with fludrocortisone. Over time, a complex deterioration of water and sodium balance emerged, involving cerebral salt-wasting syndrome, diabetes insipidus, and glucosuria. The therapeutic approach proved to be prolonged, and under a combined regimen involving fludrocortisone, desmopressin, and restricted fluid intake, a significant improvement in diuresis was observed.

In the two sequential spinal DSAs (with a 9-day interval), both cervical and thoracic, there was an absence of a vascular pathology, despite the presence of a known subdural and subarachnoid bleeding at T1/2. Subsequent follow-up MRI scans demonstrated neither signs of rebleeding nor the origin of the hematoma in the cervical and thoracic spinal regions (Figure 7E,F). However, in an abdominothoracic CTA, small bony microspurs were identified at the level of C7/T1 and more prominently at level T1/T2 (Figure 7G,H). The question remains open as to whether these bony microspurs could be implicated as the cause of the bleeding or at least to what extent they may be considered a potential contributor to the bleeding. An alternative hypothesis suggests that a bony microspur may have caused CSF (cerebrospinal fluid) accumulation analogous to a CSF fistula, ultimately leading to secondary hemorrhage. This mechanism could represent a potential etiology for spinal SAH in the absence of an identifiable vascular pathology. A surgical resection of the microspur was not performed, as we suspected that scar tissue had likely already formed. Consequently, a surgical intervention was not expected to provide any significant benefit.

The patient was discharged in a conscious and fully oriented state, demonstrating full strength in both the upper and lower extremities, and without any impairment related to bladder or rectal function. The next phase of care involves follow-up rehabilitative treatment.

During the last follow-up after four months, the clinical examination showed no signs of sensorimotor deficits. The patient exhibited only minor instability during the tandem gait test, leading to an mRS score of 1.

Table 1 presents a detailed overview of the clinical profiles of all the six cases analyzed in this study, including patient characteristics, presenting symptoms, neurological status, anatomical location of the pathology, diagnostic approaches utilized, and the therapeutic strategies implemented.

### 3.7. Literature Review

We conducted an updated literature review to incorporate the most recent cases of spinal subarachnoid hemorrhage reported between 2021 and 2024. Clinical details and diagnostic parameters, including patients’ age and sex, the specific location of the aneurysm or associated pathology, symptoms, correlation with arteriovenous malformations (AVMs) or other vascular diseases, aneurysm size, imaging techniques employed for diagnosis, treatment approaches, and neurological outcomes are summarized in Table 2.

## 4. Discussion

### 4.1. Etiology of Spinal SAH and Spinal Aneurysms

Spinal SAH can be provoked by a diverse range of causes—among these, vascular malformations and tumors contribute as the main etiological factors [36]. Spinal SAH is rarely caused by traumatic incidents [37] or by intake of oral anticoagulants (DOACs). In particular, sSAH associated with apixaban (e.g., our Case 4) was documented in only two previous cases in the literature [38,39].

It is hypothesized that minor trauma as an inciting event may trigger swift alterations in intrathoracic and intra-abdominal pressure. The subsequent rise in intraluminal pressure could lead to the rupture of small vessels within the subarachnoid space. Mechanical factors, such as spondylosis, disk herniation, arachnoiditis, or thickening of the yellow ligament, may contribute to the pathogenesis [40].

Spinal aneurysms have unique characteristics distinct from their intracranial counterparts [41]. Predominantly flow-related, these aneurysms can also manifest congenitally [8]. Spinal aneurysms frequently appear in smaller-caliber vessels along the arterial trajectory, typically away from bifurcation sites (non-bifurcation points) and show reduced susceptibility to atherosclerosis. By contrast, cerebral aneurysms tend to emerge in large-caliber vessels, particularly at bifurcation points [42]. Most spinal aneurysms exhibit diameters of less than 3 mm, with a more fusiform than saccular configuration. Dissecting aneurysms are a common occurrence, and partial thrombosis has been documented in specific cases [43,44], such as in our Case 2. The anterior spinal artery is predominantly affected, closely followed by the posterior spinal artery [45].

Earlier pathological investigations into spinal aneurysms revealed the absence of elastic fibers and the tunica media in the aneurysmal wall [8,46,47]. Conversely, some studies have pointed to the presence of fibromuscular hyperplasia or congenital irregularities in the vascular structure [8,46,47].

Spinal aneurysms can present as either isolated occurrences or in conjunction with other vascular anomalies. When unaccompanied by additional vascular abnormalities, these aneurysms are classified as isolated spinal artery aneurysms, predominantly presenting in the anterior spinal artery [45,48].

### 4.2. Clinical Presentation of Spinal SAH and Spinal Aneurysms

An extensive systematic review unveiled diverse symptom presentations in patients with spinal SAH [48]. Spinal artery aneurysms can present either as space-occupying lesions or may initially become apparent to clinicians due to rupture, leading to a syndrome of SAH that highly depends on the location of the vascular lesion within the spinal cord.

Clinical manifestations include headache and back pain, either individually or in combination with vomiting. Notably, some patients reported pure back pain, while others did not complain of headache or back pain. Various presentations, such as altered consciousness, limb weakness/paresis, and urinary disturbances, were observed. Significant variations became apparent in the manifestation of bladder involvement, particularly in cases of thoracic and thoracolumbar spinal aneurysms. Moreover, patients with arteriovenous malformation-associated spinal aneurysms exhibited a higher likelihood of spinal cord dysfunction, including myelopathy and sensory findings, compared to those with isolated spinal aneurysms [48]. Furthermore, the presence of concurrent meningeal signs (e.g., neck stiffness/nuchal rigidity, photophobia) and abdominal pain could serve as indicative signs of sSAH [49,50].

The clinical presentation of spinal aneurysms poses challenges, as symptoms may range from headaches to abdominal or neurological symptoms.

### 4.3. Imaging Modalities

The preferred diagnostic modality for sSAH and spinal aneurysms is magnetic resonance imaging (MRI), with subsequent confirmation by spinal angiography. In cases where a substantial amount of hemorrhage is present, potentially obscuring its origin, it is advisable to consider short-term follow-up MR imaging (within 1–2 weeks). For focal lesions, spinal aneurysms should be considered among potential causes, warranting angiography for further evaluation. Even if angiography does not detect a vascular pathology, the possibility of a thrombosed aneurysm should still be considered. Further vascular imaging of the spine is recommended (e.g., CT or MR angiography) in cases of negative initial DSA, particularly when there is a notable high blood volume observed on CT around the brainstem or upper cervical cord [22]. A CT angiogram of the head and neck at follow-up could be beneficial, as an anterior spinal artery aneurysm in one case report was detected in a subsequent CT angiography conducted three months after the initial presentation [17]. Additionally, when confronted with aneurysms of small dimensions or partially thrombosed aneurysms, it becomes challenging to localize the exact origin of the hemorrhage, necessitating a multimodal sequential imaging approach [6].

### 4.4. Treatment of Spinal SAH and Spinal Aneurysms

The management of spinal artery aneurysms remains a topic of debate. Three approaches have been taken into consideration: endovascular treatment, surgical intervention, and conservative therapy [30,50,51].

Microsurgical techniques, such as clipping or resection, and endovascular treatments are indicated, particularly when dealing with dorsally or dorsolaterally located aneurysms, achieving favorable results [45].

In the presence of symptomatic cord compression, prompt surgical intervention is essential to preserve or improve neurological function [21]. However, in cases where urgency is not indicated, management decisions can be influenced by factors such as origin, artery size, distal flow, morphology, and location of the lesion [51]. An early surgical approach is recommended for posterior spinal aneurysms due to their easily accessible and safely resectable nature [52]. By contrast, anterior spinal aneurysms pose a greater therapeutic challenge due to their intricate accessibility and significant role in supplying blood to the spinal cord [24,53].

Opting for conservative management stands as a viable option when maintaining blood flow through the vessel is crucial to prevent ischemic episodes. Clinicians should take various factors into account to develop an appropriate treatment strategy tailored to the individual vascular anatomy.

Before 2020, spinal aneurysms were rarely documented, with only a few case reports detailing aspects such as patient demographics, aneurysm location, symptoms, size, imaging techniques, treatments, and neurological outcomes [17,21,23,24,54]. This highlights the need for further research in this area. Only one review meticulously gathered cases through exhaustive and systematic searches across various databases [42]. In light of this, we updated a literature review to encapsulate the most recent cases reported between 2021 and 2024 (Table 2). Unlike earlier studies that focused on cases before 2021, our research incorporates recent data, offering a broader perspective by addressing multiple etiologies of spinal SAH, including aneurysm rupture, direct oral anticoagulants, and bony microspurs. This multifaceted approach, combined with detailed case evaluations and multimodal imaging, highlights the complexity of spinal SAH, emphasizes individualized management, and provides a novel contribution to the field.

However, several limitations should be acknowledged to contextualize the findings and guide future research. The study’s cohort included only six patients from a single center diagnosed with sSAH over a four-year study period. While spinal subarachnoid hemorrhage is an exceedingly rare condition, the limited sample size constrains the generalizability of the results and the ability to derive robust statistical inferences. Larger, multicenter studies are needed to validate these findings, and controlled cohorts could clarify the efficacy of these approaches. 

## 5. Conclusions

In conclusion, identifying the cause of sSAH is critical for effective management, as clinical presentation, treatment strategies, and outcomes vary based on the aneurysm’s pathology and location. Three patients with sSAH due to spinal artery aneurysms were accurately diagnosed and treated, but other causes (anticoagulation, microspurs, or other underlying diseases) should be considered. With limited literature available, our study highlights the need for a personalized approach to the diagnosis and treatment of spinal subarachnoid hemorrhage.

## Figures and Tables

**Table 1:** Patient characteristics, symptoms, neurological status, location of pathology, diagnostic measures, and therapies.

Case	Sex	Age	Symptoms	Initial Neurological Status	Location of the Aneurysm/SAH	Etiology	Diagnostic Measures	Therapy
1	F	47	headaches and pain in the back/neck pain, nausea, vomiting, photophobia/phonophobia	no focal neurological deficit	SAH Fisher IV with spinal aneurysm at T1	aneurysm	lumbar puncture, multiple DSA, multiple spinal MRI	conservative
2	F	69	headaches and abdominal complaints	oriented 3×, marked meningism, positive Lasègue sign	spinal SAH with (partial) thrombosed intraspinal aneurysm at T2	aneurysm	multiple DSA, multiple spinal MRI	surgical procedure (resection of the aneurysm)
3	F	25	acute headaches, complete tetraplegia, bradypnea, and asystole → cardiopulmonary resuscitation with ROSC	hypoesthesia on the left side at C6 and left thoracic and abdominal areas, left hemiparesis, tremor, and hemiataxia	spinal intramedullary aneurysm at C2 with perimedullary fistula	aneurysm	multiple DSA, multiple spinal MRI	surgical procedure (arterial feeder coagulation)
4	F	86	iliosacral pain, paraparesis of the legs, and hypoesthesia from T11	no evidence of manifest paralysis, intact sensation, unavailable reflexes in the lower extremities	spinal SAH; initially suspected T11 aneurysm; angiographically, no evidence of an aneurysm (spot sign)	anticoagulation (apixaban)	2 lumbar punctures, repeated abdominal/thoracic CTA, DSA	conservative
5	F	73	headaches and pain in the back/neck pain	pronounced paraparesis of both legs	spinal SAH; angiographically, no evidence of an aneurysm	anticoagulation (rivaroxaban)	abdominal/thoracic CTA, multiple DSA, multiple spinal MRI	surgical procedure (evacuation of an intradural hematoma)
6	M	55	pain between the shoulder blades and the cervical spine, tingling sensations in all four extremities	tingling sensations in all extremities, unable to lift the legs, weakness in both arms	epidural bleeding and spinal SAH; suspected perimedullary aneurysm at T1/2; angiographically, no evidence of an aneurysm, epidural bleeding may be due to a microspur at T1/2	microspur	multiple DSA, multiple spinal MRI	surgical procedure (evacuation of an epidural hematoma), then conservative (no evidence of an aneurysm)

CTA: CT angiography; DSA: digital subtraction angiography; F: female; M: male; MRI: magnetic resonance imaging; ROSC: return of spontaneous circulation; SAH: subarachnoid hemorrhage.

**Table 2:** Review of the literature (recent reported cases of spinal subarachnoid hemorrhage and spinal artery aneurysms, 2021–2024).

Authors	Age	Sex	Location of the Aneurysm/Pathology	Symptoms	Association with AVMs or Other Associated Vascular Disease	Size	Imaging Modality	Treatment	Neurological Outcome
Kawai et al., 2021 [16]	83	M	posterior fossa	unconsciousness	yes (bilateral vertebral artery occlusion)	6 × 5 mm	CT, MRI, DSA	endovascular (coil embolization)	bed-ridden
Abdalkader et al., 2021 [17]	70s	M	anterior spinal artery C2	headache, vomiting	yes (bilateral vertebral artery occlusion)	3 × 2.5 mm	cCT, CTA	surgery (clipping)	stable neurological status
	60s	F	T3/T4	neck and shoulder pain, headache	no	6 × 3 mm	cCT, CTA, MRI, DSA	conservative treatment	stable neurological status
	50s	M	C2/C3	neck pain, headache, vomiting	no	5 × 2.5 mm	CTA, MRI	conservative treatment	stable neurological status
	40s	F	C1/C2	confusion, decreased level of consciousness	no	3 mm	cCT, CTA, DSA	conservative treatment	death (severe diffuse vasospasm, bihemispheric infarcts, cardiopulmonary arrest)
Bergeron et al., 2021 [18]	56	F	posterior spinal artery T11	thunderclap headache, sudden cervical pain, vomiting, leg pain	no	5 mm	cCT, MRI, DSA	surgery (excision)	stable neurological status
	51	F	T5, T7	thunderclap headache, nausea, vomiting, weakness of the right leg	no	5 mm (T5), 3.5 mm (T7)	cCT, CTA, MRI, DSA	conservative treatment	stable neurological status
	72	F	T10	headaches, dorsolumbar pain, vomiting, severe back pain, nuchal rigidity, left proximal leg weakness	no	4 mm	cCT, MRI, DSA	conservative treatment	left leg weakness remained unchanged
	37	F	T3	cervical, thoracic pain, headache, neck stiffness	no	8 mm	cCT, MRI, DSA	conservative treatment	stable neurological status
Cadieux et al., 2021 [19]	54	F	T2	headache, intrascapular pain, acute paraplegia	yes (hypophyseal artery aneurysm)	4 mm	cCT, CTA, MRI, DSA	surgery (1. cervicothoracic decompression and hematoma evacuation; 2. resection of the aneurysm)	near-complete return to normal strength
Duangprasert et al., 2021 [20]	71	F	right anterior spinal artery arising from the vertebral artery	severe headache, hemiparesis	no	3.1 mm	cCT, CTA, DSA	surgery (occipital artery–PICA bypass)	mRS score = 1
Tenorio et al., 2021 [21]	49	N/A	posterior spinal artery T11/T12	walking difficulty, diaphoresis, back and abdominal pain, and paraplegia	no	1.7 cm (intradural cystic mass)	MRI, DSA	surgery (excision)	partial recovery
Limaye et al., 2021 [14]	43	F	T12	severe lower back pain, paresthesias in the lower extremities	no	4 × 2 mm	cCT, MRI, DSA	conservative treatment	no neurologic deficits
Malhotra et al., 2021 [22]	76	F	posterior spinal artery T10/11	lower back pain, headache, nuchal rigidity, depressed level of consciousness	no	7 × 4 mm	cCT, CTA, MRI, DSA	endovascular (glue embolization)	mildly impaired tandem gait
Shima et al., 2021 [23]	77	F	C3	headache, nausea, hemiparesis, disturbance of consciousness	yes (bilateral vertebral artery occlusion)	3 mm	cCT, CTA, DSA	endovascular (coil embolization)	mRS score = 3
Turrini et al., 2021 [24]	64	M	C1	dizziness, numbness, headache, vomiting	no	N/A	cCT, MRI, DSA	surgery (excision)	gradual recovery after rehabilitation
Watanabe et al., 2021 [25]	78	M	C3	severe headache, slight hemiparesis	yes (bilateral vertebral artery occlusion)	8 mm	cCT, MRI, DSA	endovascular (parent artery occlusion of contralateral vertebral artery aneurysm)	death
Chen et al., 2022 [26]	68	F	C2	headache, lethargy, nuchal rigidity	no	approx. 3 mm	cCT, DSA	unsuccessful endovascular procedure, surgery (clipping)	death (sudden cardiac arrest)
Crobeddu et al., 2022 [27]	62	M	L1	paresthesia in the lower limbs and walking impairment	no	N/A	MRI, DSA	surgery (decompressive hemilaminectomy due to spinal cord compression), conservative treatment (spontaneous occlusion)	fully recovered after surgery
Kulubya et al., 2022 [28]	49	F	C1	sudden-onset severe headache and nausea	no	1 × 1 × 1.3 mm	cCT, DSA	surgery (wrapping)	no neurologic deficits
Otaki et al., 2022 [29]	54	M	left vertebral artery—anterior spinal artery bifurcation	not reported (severe SAH WFNS grade II)	no	2 mm	cCT, DSA	endovascular (coil embolization)	mRS Score = 0
McGuire et al., 2023 [30]	23	M	T7	neck pain, paraplegia	no	N/A	MRI, DSA	endovascular (glue embolization); surgery (hematoma evacuation)	good neurological status (ambulatory)
	54	M	L3	headache, back pain, leg pain, lower extremity weakness	no	N/A	MRI, DSA	endovascular (glue embolization), surgery (hematoma evacuation)	good neurological status (ambulatory)
	72	F	T12	neck pain, back pain, paraplegia	no	N/A	MRI, DSA	surgery (trapping with hematoma evacuation)	good neurological status (ambulatory)
	60	F	T3, T6, T10	lower extremity weakness	no	N/A	MRI, DSA	conservative treatment	good neurological status (ambulatory)
	64	M	T8, T10	back pain, urinary retention	no	N/A	MRI, DSA	conservative treatment	good neurological status (ambulatory)
Zanaty et al., 2023 [31]	76	F	AVM at T9/10	unsteady gait, thoracic pain, numbness, weakness in both legs, urinary retention	yes (AVM)	N/A	MRI, DSA	surgery (clipping, AVM resection)	recovery of the bladder and motor function, impaired proprioception
Sokol et al., 2024 [32]	58	M	pseudoaneurysm C5/C6	neck pain, headache, mild upper extremity ataxia	no	1 mm	cCT, MRI, DSA	conservative treatment	mild balance difficulties
Zhou et al., 2024 [33]	74	N/A	T2	back pain	no	N/A	MRI	surgery (parent artery sacrifice)	mRS score = 1
	32	N/A	T1	headache	yes (coarctation of the thoracic aorta)	7.53 mm	DSA	conservative treatment	mRS score = 0
	34	N/A	C7	headache	yes (coarctation of the thoracic aorta)	3.64 mm	DSA	endovascular (coil)	mRS score = 0
	16	N/A	C2	headache	unclear (multiple aneurysms may indicate connective tissue disease)	2.10 mm	DSA	endovascular (coil)	death (due to SAH 5 months post-discharge)
	29	N/A	T12	lower back pain, lower extremity weakness, urinary retention	unclear (vestige of arteriovenous shunt suspected)	3.03 mm	DSA	conservative treatment	mRS score = 1
	57	N/A	C6	headache	no	4.24 mm	DSA	conservative treatment	mRS score = 0
	57	N/A	T12	lower back pain, lower extremity weakness, urinary retention	no	N/A	MRI	surgery (parent artery sacrifice)	mRS score = 3
Ioannidis et al., 2024 [34]	37	F	T8	acute headache, vomiting, episode of loss of consciousness, 3/5 right leg paresis	yes (AVM)	12 × 11 mm	cCT, CTA, DSA	endovascular (coil)	stable neurological status
Ahmadpour et al., 2024 [35]	30	M	anterior spinal artery T11/T12	acute-onset back pain and bilateral lower extremity motor paraplegia	yes (infectious etiology assumed: mycotic aneurysm)	7 × 5 mm	cCT, MRI, DSA	conservative treatment	death (complications from severe sepsis secondary to necrotizing pneumonia attributed to the patient’s underlying autoimmune disease and thrombotic microangiopathy)

## Data Availability

Authors can confirm that all relevant data are included in the article. The dataset(s) was/were derived from public resources and made available in the article (references).
